# Supplementary figures and images for: Proteomic and ubiquitinome analysis reveal that microgravity affects glucose metabolism of mouse hearts by remodeling non-degradative ubiquitination
Source: PLoS One. 2024 Nov 14;19(11):e0313519. doi: 10.1371/journal.pone.0313519 (PMC11563481; doi:10.1371/journal.pone.0313519)

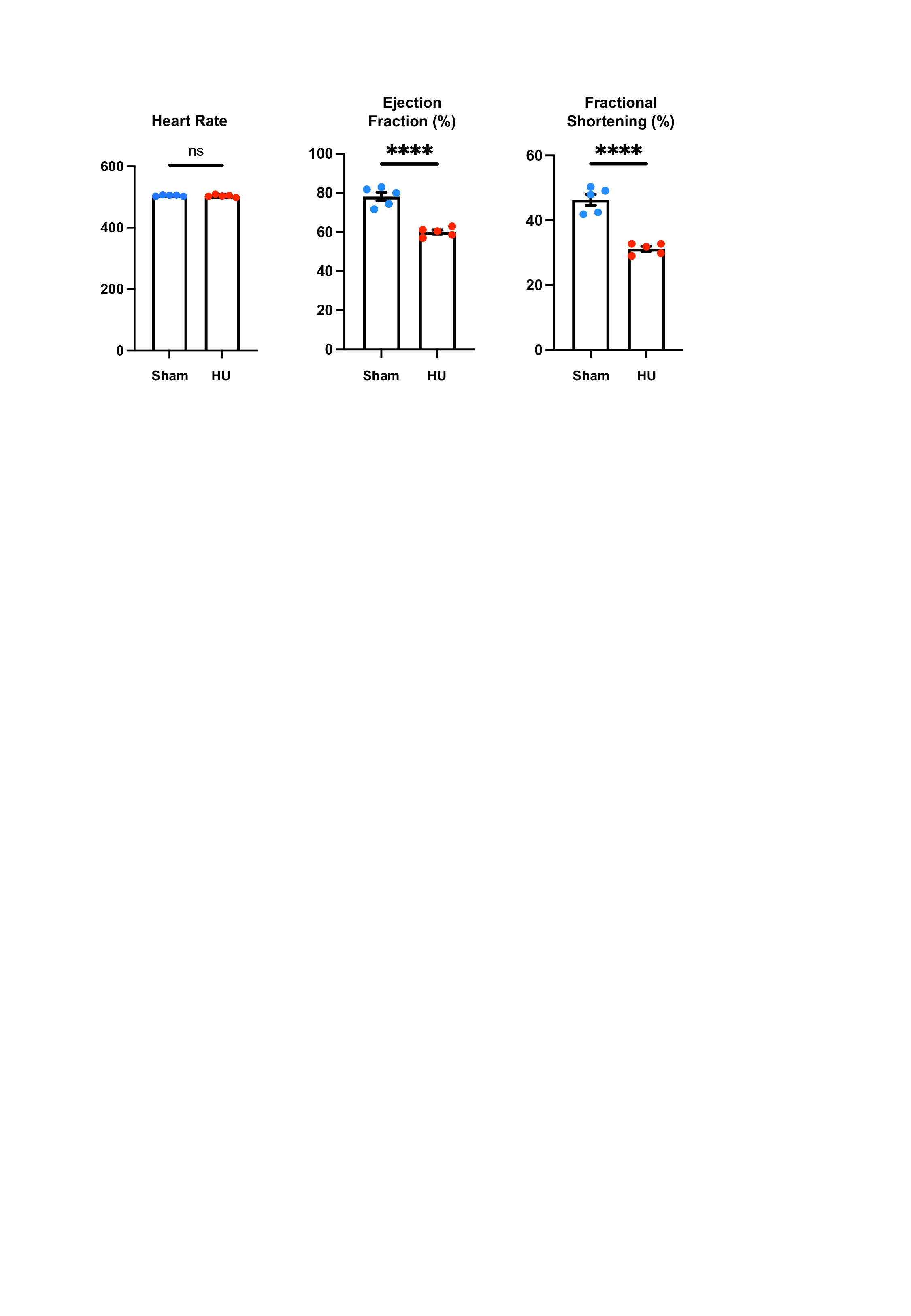

Supplement: S1 Fig — Utilizing echocardiography, measurements of left ventricular dimensions in both systole and diastole were meticulously performed for the mice in the sham group as well as those in the hindlimb unloading (HU) group. Subsequently, the ejection fraction (EF) and fractional shortening (FS) were calculated based on these measurements. n = 5 mice per group. Data were presented as mean ± SEM. ****p < .0001. (TIF) [file pone.0313519.s001.tif]

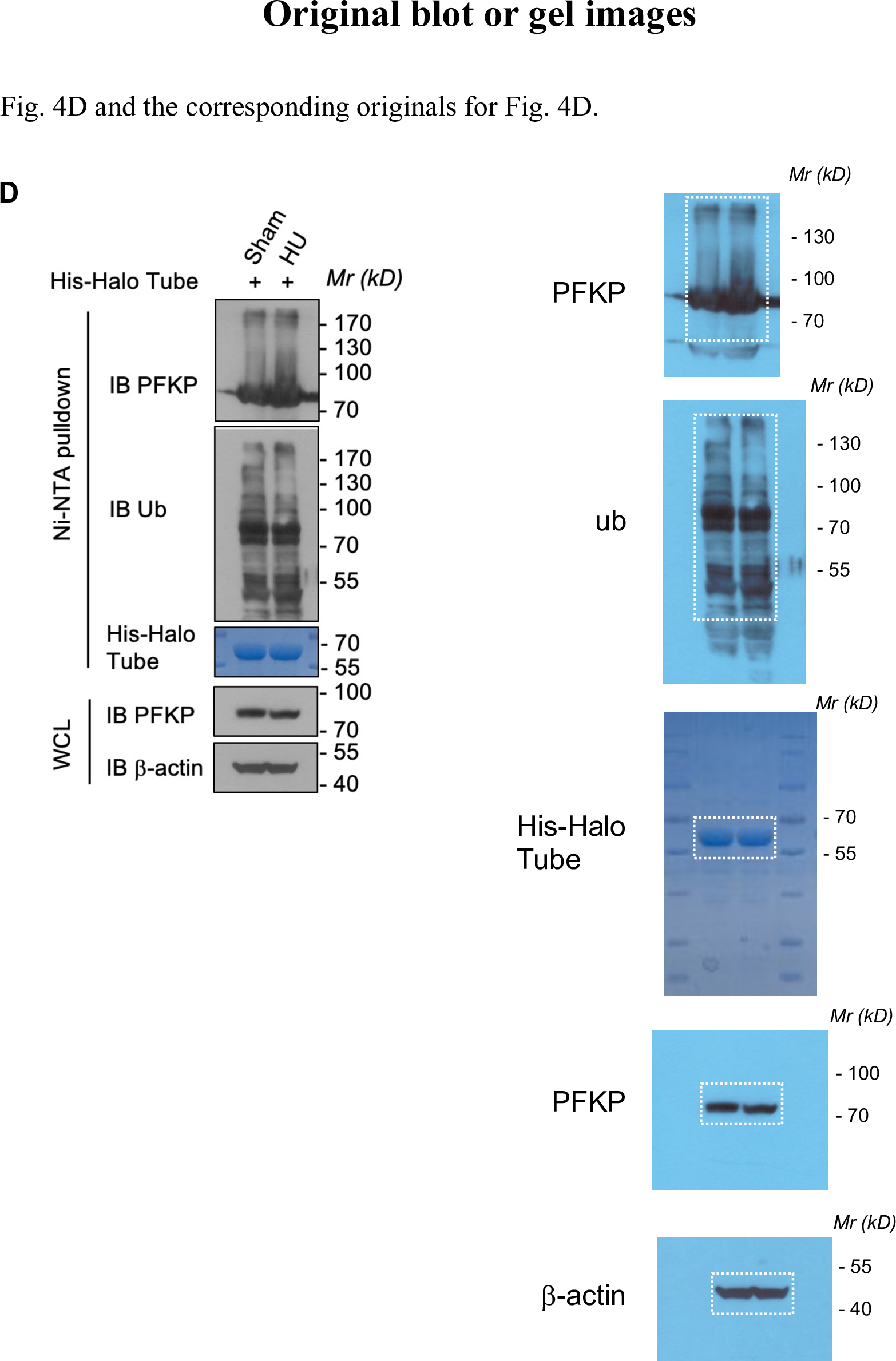

Supplement: S1 Raw image — (TIF) [file pone.0313519.s007.tif]
