# Supplementary material for: Proteomic and ubiquitinome analysis reveal that microgravity affects glucose metabolism of mouse hearts by remodeling non-degradative ubiquitination
Source: PLoS One. 2024 Nov 14;19(11):e0313519. doi: 10.1371/journal.pone.0313519 (PMC11563481; doi:10.1371/journal.pone.0313519)
Supplement: Attachment — Submitted filename: 3-Response to Reviewers edited.docx [file pone.0313519.s009.docx]

**Response letter**

Point-by-point responses to the referees’ comments

RE: Zhang et al., PONE-D-24-28980, “Proteomic and ubiquitinome analysis reveal that microgravity affects cardiac functions by remodeling glucose metabolism”.

1. Methodological Limitations: While the study uses proteomic and ubiquitinomic analyses, it fails to account for potential confounding variables in the experimental setup. For instance, the tail suspension model used to simulate microgravity is well known but has limitations in accurately mimicking the conditions experienced in space. This raises concerns about the validity and translatability of the findings to actual spaceflight conditions. The authors should address how they intend to mitigate these limitations or at least provide a more robust discussion of the model’s limitations.

**Response: We sincerely appreciate the suggestions given by the editor. In response to this issue, we have added "Limits of the study" at the end of the "Conclusion" section of the article to supplement the explanation of the limitations of this model and elaborate on how to alleviate these limitations in the future.**

2. Data Presentation and Clarity: The figures and tables provided, while comprehensive, are often confusing. For example, Figure 1 displays proteomic analysis but lacks sufficient annotations for non-expert readers to fully understand the significance of the data. The legends accompanying the figures are vague and do not provide enough information regarding statistical analyses or controls used. This weakens the overall impact of the presented data and calls into question the validity of the results.

**Response: We are very sorry that the legend was not expressed clearly enough. We have made modifications in these legends.**

3. Lack of Contextual Integration: The authors discuss cardiac dysfunction in a microgravity environment but fail to integrate similar relevant studies effectively into the introduction. For example, recent findings such as those by Al-Awaida et al. (2020, 2023) on the effects of simulated microgravity on the biological properties of wheatgrass and its therapeutic effects on diabetes and breast cancer under microgravity conditions could provide valuable context. These studies have demonstrated that microgravity can significantly modulate metabolic and physiological responses in mammalian models. Including such literature would greatly enhance the introduction by providing a broader perspective on how microgravity affects not only cardiac function but also other biological systems.

**Response: We are very grateful for the suggestions given by the editor.** **We have added some background content in the introduction section and integrated some related researches to emphasis the impacts of microgravity.**

4. Inadequate Interpretation of Ubiquitinome Results: The interpretation of the ubiquitinome data lacks depth. While the authors claim that non-degradative ubiquitination plays a role in metabolic dysfunction, they do not provide sufficient mechanistic insights or experimental validation to support this claim. For instance, the observed changes in kinase activity related to glucose metabolism are not fully explained in terms of their physiological relevance or implications for cardiac dysfunction. Furthermore, there is little discussion on how these findings could inform therapeutic interventions or countermeasures for cardiac atrophy under spaceflight conditions.

**Response: We are very grateful for the suggestions given by the editor.**

**1) Our results suggested an unexpected activation of PGK1, PFKP, and ALDOA by non-degradative ubiquitination under weightless conditions in hearts. Previous studies have reported that excessive activation of glucose metabolism interferes with normal metabolic signaling pathways, affects cardiomyocytes metabolism, and finally damages heart function (****Physiol Rev, 2005; Nat Rev Cardiol, 2018; Circ Res, 2020). Therefore, our data indicated that the activation of PGK1, PFKP, and ALDOA by non-degradative ubiquitination under weightless conditions led to metabolic disorders in hearts, and thus provided a potential explanation for microgravity-induced cardiac dysfunction.**

**2) We have provided more discussion in the revised manuscript. At present, some compounds, such as 3-BrPA, significantly inhibit the glycolytic activity, attenuate pulmonary artery remodeling and ventricular hypertrophy, thereby improving heart function (Am J Hypertens, 2019). This suggests that drugs targeting glucose metabolism would be potential drugs for the recovery of microgravity-exposed hearts, worthy of further study and test for cardiac atrophy under spaceflight conditions in the future.**

5. Conclusions Exceed the Evidence: The conclusions drawn in the paper are overly broad and do not sufficiently align with the data presented. Specifically, the claim that the study provides "valuable insights for long-term human space exploration" is premature, given the limitations in experimental design and lack of direct spaceflight data. The authors need to scale back their conclusions and provide a more conservative interpretation of their findings until they are validated by further research, possibly in actual spaceflight conditions.

**Response: We are very sorry that our conclusion is too broad, and we also greatly appreciate the suggestions given by the editor. We have modified the statements in the Introduction, and also discussed the limitations of the experimental design in the "Limits of the study" section.**

**Response to Reviewers' Comments：**

We sincerely thank the two reviewers for their evaluation of this paper and their recognition of it. Below are our responses to the reviewers' questions.

Reviewer #2: Hello,

Thanks for submitting manuscript titled "Proteomic and ubiquitinome analysis reveal that microgravity affects cardiac functions by remodeling glucose metabolism" to this journal.

1. It is an interesting article trying to assess the same in MICE and not humans. No where in either the title or abstract this is clarified as to what subgroup of population was evaluated. Though the proteins changes can be extrapolated across the species but the proof of the same lies on the investigator and hence it would be important to mention Mice derived proteins rather than a blanket statement.

2. The methods and ethical considerations needs to be elaborated further in the manuscript.

3. there are minor grammatical and typographical errors which need to be rectified by a native English speaking subject expert.

Otherwise, it is a well written draft and can be accepted after the revision.

Thanks

**Response: We are very grateful for the valuable suggestions put forward by the reviewers. We have modified the title, supplemented and improved the methods and ethics in the** **manuscript, and also re-proofread the manuscript. We sincerely appreciate your recognition of this research.**
